# Exploring the Accessibility of Community-Based Telerehabilitation for Children with Disabilities from Low-Income Households

**DOI:** 10.5195/ijt.2024.6625

**Published:** 2025-01-15

**Authors:** Karen S. Sagun, Ryanne Nicole H. Alava, Kristine Therese S. Cablay, Katelyn A. Dagdag, Francis Rowelle P. Lagman, Kvaern Edgar S. Nocos, Jamela Y. Quidilla, Nina Mari M. Tan

**Affiliations:** 1 Occupational Therapy Department, College of Rehabilitation Sciences, University of Santo Tomas, Manila, Philippines; 2 Quezon City Kabahagi Center for Children with Disabilities, Quezon City Government, Philippines

**Keywords:** Children with Disabilities, Community-Based Rehabilitation, Philippines, Telerehabilitation, Urban poor

## Abstract

Community-Based Rehabilitation (CBR) is a rights-based approach that aims to provide equitable health services and participation opportunities for people with disabilities. Telerehabilitation has emerged as a potential methodology for delivering health care within the CBR framework. However, the accessibility of telerehabilitation presents unique challenges for children with disabilities (CWD) in communities with low socioeconomic status. This phenomenological qualitative study explores the barriers and facilitators that influence the participation of families of CWD in telerehabilitation as a method of CBR in urban poor communities. Nine focus group discussions (FGDs) were conducted involving 75 primary caregivers of CWD. Inductive thematic analysis using NVivo software was employed to analyze the collected data, revealing four themes that impact participation in Telerehabilitation: Economic and Social Resources, Self-Competency Affecting Transition, Flexible Service Delivery Mechanism, and Safety and Security as a Threat to Participation. The findings emphasize the intricate interplay of factors influencing the accessibility of telerehabilitation services for CWD from low-income households. Actionable strategies to address these challenges include providing affordable technology and internet access, offering training and support to families, developing culturally sensitive resources, establishing safety protocols, and advocating for inclusive policies and funding mechanisms. Collaboration among healthcare professionals, policymakers, and families is essential in building a resilient and equitable healthcare system that prioritizes the needs of CWD living in poverty. The insights gained from this study can inform the development of targeted interventions and support systems to ensure that no child is left behind in accessing quality care despite the digital and socioeconomic divides.

Community-Based Rehabilitation (CBR) is a strategy that ensures people with disabilities (PWD) have equal rights and opportunities in healthcare, education, and skills training within their communities (World Health Organization, 2015). The Twin Track approach in CBR practice addresses the specific needs of PWD while promoting inclusive community development (Madsen et al., 2021). CBR is relevant for communities experiencing environmental barriers, economic restrictions, resource and service limitations for PWDs (Lukersmith et al., 2013). In the Philippines, an estimated 325,000 children are identified with a disability card, representing one out of five children with disability (CWD) from a total of 1.27 million (UNICEF, 2022). CBR programs increase the accessibility of existing institutional rehabilitation services and local resources in urban low-income areas (Robertson et al., 2012). Through CBR, CWDs gain knowledge and better understand their disability while experiencing increased involvement and support from family members, reduced feelings of isolation, decreased dependence on others, and a transformation from passive to active community members (Bongo et al., 2018).

## Transition to Remote Methods due to Public Health Emergencies

The public health emergency has significantly impacted routine healthcare services, including therapy services for CWD, due to reduced capacity and temporary halts in some establishments (Kubenz & Kiwan, 2022). As a result, therapy services have shifted to telerehabilitation, which involves delivering health-related services through information and communication technologies (ICT) when the healthcare provider and client are in different physical locations or when in-person engagements are not possible or practical (World Federation of Occupational Therapists, 2021). Telerehabilitation can be delivered synchronously, consisting of real-time interactions with clients, or asynchronously, involving data transmission between the therapist and client.

### Methods of Doing CBR in Low Resource Settings

Due to the limited in-person services caused by public health emergencies such as COVID-19, several methods of conducting CBR in low-resource settings have been adapted to provide therapeutic services. These methods comprise synchronous and asynchronous modes of communication. Synchronous modes include telephones, messaging, emails, video conferencing tools, and other interactive web-based platforms that enable cost-effective and flexible virtual meet-ups, crucial for safety measures during public health emergencies (Ownsworth et al., 2019). Asynchronous modes include video clip demonstrations, video return demonstrations, consultations through photos with text explanations, feedback via preferred platforms, and phone call consultations to facilitate individual and self-paced learning and reduce instructor dependence (Fabriz et al., 2021).

### Family-Centered Practice

A stay-at-home setting for telerehabilitation services may benefit from a participatory approach that considers every stakeholder's perspective. This approach promotes the concept of family-centered practices (FCP), in which professionals acknowledge that parents are the key persons who know their children best and that optimal child functioning occurs through family and community support (Kolehmainen et al., 2010). FCP can facilitate empowerment for the parents (Kruijsen-Terpstra et al., 2016). Recognizing the family's significance as the primary educator, supporter, and caregiver is crucial in treating the client within the family context.

### Experiences of Parents in Transitioning to Telerehabilitation

As health services offer telerehabilitation for remote service provision as recommended by WHO, CWDs are likely to face several barriers and reduced access to digital technology or remote appointments (Pregel & Le Fanu, 2020). This is more common in low to middle-income countries, such as the Philippines, and may significantly affect people in rural or remote locations (Kubenz & Kiwan, 2022). This calls for examining parents' experiences and needs during the shift to telerehabilitation.

Most parents in low-resource settings were hesitant about undergoing training using an online platform (Sengupta et al., 2021). They expressed not being “professionals” since they have inadequate knowledge to confidently implement the strategies themselves (Eguia & Capio, 2022). This shift has also placed additional roles and responsibilities on parents. Many struggled with balancing employment demands while supporting their child's needs and tending to other children in the household (Tambyraja et al., 2021). Online therapy is seen as time-consuming, taking parents' time away from other things, such as self-care (Murtonen, 2018). Technical difficulties were frequently reported and described as a source of frustration (Ashburner et al., 2016). Furthermore, challenges faced with children's behavior, such as inattention, distractibility, difficulty adapting, task avoidance, and tantrums, were significantly raised (Delos Reyes et al., 2021).

### Conceptual Framework

Telerehabilitation has become an increasingly crucial component within the CBR framework. This study aims to investigate the factors influencing the participation of CWD and their families from low socioeconomic backgrounds in telerehabilitation programs. The research objective was to describe barriers and facilitators this population encounters, which affect their access to and engagement in telerehabilitation services.

**Figure 1:**
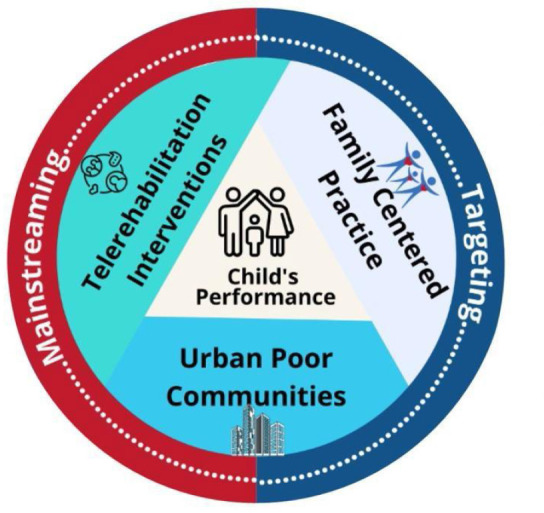
Conceptual Framework

This study utilized a conceptual framework (Figure 1) that explored the complex relationships between economic factors, family dynamics, and healthcare accessibility in the context of virtual therapy services. Understanding these interconnections helps identify specific barriers and facilitators faced by families from low socioeconomic backgrounds, ultimately informing the development of targeted policies, programs, and resource allocation strategies. This research aims to contribute to the mainstreaming of telerehabilitation by ensuring more equitable access to services for this vulnerable population.

Moreover, the insights gained from this research inform healthcare providers about the diverse needs of CWD and their families from low socioeconomic backgrounds. This understanding will encourage the adoption of inclusive, family-centered practices that recognize and respond to the specific challenges faced by this population. By fostering a supportive ecosystem that addresses the barriers identified in the study, healthcare providers can work towards ensuring equal access to quality care for all CWD, regardless of their socioeconomic status.

## Methodology

### Research Design

This study employed a qualitative phenomenological research design to explore the perceived barriers and facilitators experienced by families of CWD regarding remotely implemented Community-Based Telerehabilitation. Qualitative research utilizes a wide array of rigorous study approaches and techniques to gain an understanding of peoples’ experiences and opinions as well as reasons and motivations for behaviors (Bazen et al., 2021), and allows participants to express their feelings, thoughts, and experiences related to telerehabilitation. Phenomenological research design enables researchers to describe the essence of a phenomenon by eliciting meaning from the perspective of the contextualized experiences of individuals (Neubauer et al., 2019). Focus Group Discussion (FGD) is a form of group interview facilitated by a moderator, focusing the discussion on a specific topic (Doody et al., 2013). Online FGDs utilize virtual space for communication through video conferences, adapting traditional methods for data collection (Nyumba et al., 2018). Considering the restrictions on travel and community mobility, online FGDs were deemed the most feasible approach to minimize risk while allowing participants to communicate freely with the researchers in real time.

### Study Site and Participants

**Figure 2:**
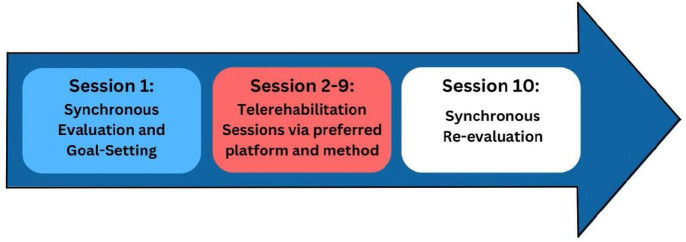
Telerehabilitation Program Process in Kabahagi Center for CWD

The participants were recipients of the telerehabilitation program provided by the Quezon City *Kabahagi Center for Children with Disabilities* in the Philippines. Quezon City, the most populous city in the Philippines with 3.1 million residents (Philippine Statistics Authority, 2013), hosts the *Kabahagi* program (Filipino word meaning “to be a part of”), which aims to promote the inclusion of CWD by providing free services, including telerehabilitation, to address the needs of CWD in urban poor communities (Montemayor, 2018). *Kabahagi Center for Children with Disabilities* has implemented a ten-session protocol using blended remote strategies (Figure 2)

Purposive sampling was used to select individuals with relevant experiences related to the phenomenon of interest, leading to the collection of meaningful information (Cresswell & Plano, 2011). Participant inclusion criteria were: (1) screened as an indigent resident of Quezon City, Philippines; (2) CWD must have a Medical Certificate verifying their diagnosis and have completed the telerehabilitation program; and (3) ability to understand and speak Filipino. Participants were excluded if the CWD did not complete the 10-session protocol. Willing participants were divided into groups of six to eight members per FGD based on their preferred schedule. This group size is beneficial since sensitive or personal experiences may be tackled during the discussions. It would also give the members enough time and opportunities to share their perspectives (Stalmeijer et al., 2014).

Nine (9) FGDs were conducted, enjoining 75 family members through Zoom meetings, with each FGD lasting for approximately 60 to 90 minutes.

**Table 1:** Parent Participants

Parent participants	Male	Female
(n=75)	5	70
**Age range**		
28–38	4	38
39–49	-	18
50–60	1	10
61–65	-	4

**Table 2:** Children of Participants

Child number	Male	Female
n = 77	46	31
**Age range (2–21)**		
2–6	19	14
7–11	22	12
12–16	3	2
17–21	2	3
**Diagnosis**		
Autism Spectrum Disorder	20	9
Cerebral Palsy	6	8
Down Syndrome	4	4
Global Developmental Delay	4	3
Intellectual Disability	3	4
Attention Deficit Hyperactivity Disorder	3	1
Becker's Dystrophy	1	-
Cerebellar Tumor	1	-
Hydrocephaly	1	-
Language Impairment	2	1
Microcephaly	1	-
Arthrogryposis	-	1

### Data Collection

The researchers obtained approval from the Ethics Review Committee of the University of Santo Tomas College of Rehabilitation Sciences. Recruitment began with distributing participant information sheets via email and phone calls, detailing the duration, overview of events, benefits, risks, and assurance of personal data privacy and confidentiality, as well as support for participation via the provision of data allowance. No financial remuneration was given to the participants during the study. Participants signed and returned consent forms via email. For those unable to send consent forms electronically, verbal consent was obtained and recorded through phone calls (Magnusson et al., 2016).

An FGD guide with open-ended questions was developed to explore families' perceptions and experiences of remotely implemented CBR through telerehabilitation. The guide was pilot-tested and revised based on the results and discussions from studies by Ashburner et al. (2016) and Dew et al. (2012). Online FGDs were conducted until data saturation was achieved, indicating that no new insights or information were being generated (Houghton et al., 2013). Note-taking and video recording were employed during each FGD. The Zoom platform was used for its meeting encryption and locking features, ensuring the security of information gathered from participants (Archibald et al., 2019).

Rigor and reflexivity were ensured throughout the study. Credibility was established by recruiting diverse participants and providing multiple perspectives. An audit trail was made, and an external transcriber was engaged to minimize bias (Maher et al., 2018).

### Data Analyses

**Figure 3:**
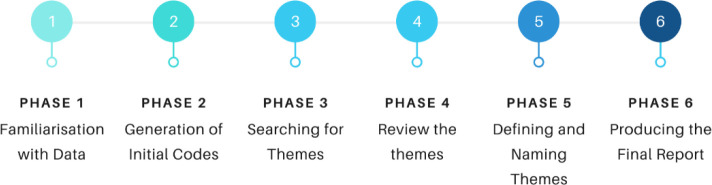
Thematic Analysis Process

Thematic analysis was employed to identify, analyze, and interpret patterns within and across transcribed data related to participants' perspectives, behaviors, and practices (Clarke & Braun, 2016). An inductive approach was used, deriving themes from descriptions of raw data based on individuals' lived experiences (Azungah, 2018). Investigator triangulation was applied, with researcher subteams analyzing the data independently to ensure consistent conclusions and enhance validity while minimizing bias (Carter et al., 2014). The 6-phase method for thematic analysis by Clarke and Braun (2016), as cited by Butchart et al. (2019), was followed (Figure 3).

The process began with (1) familiarization with the data through transcript reading, followed by (2) generation of initial codes through line-by-line encoding of nodes for systematic coding. Once a comprehensive list of codes was generated across the dataset, (3) a search for themes was conducted, categorizing and sorting different nodes into potential themes. The themes were then (4) reviewed for interconnections and (5) named and defined, accompanied by a narrative related to the guiding research objectives. Finally, (6) the report was drafted to present the findings and analysis.

## Results

Four major themes emerged in the analysis of nine focus group discussion transcripts as factors affecting participation in telerehabilitation for indigent families of CWD. See Table 3. These themes encompass the Economic and Social Resources that Support or Hinder Participation, Self-competency Affecting Transition, Flexible Service Delivery Mechanisms, and Safety and Security as a Threat to Participation. Each central theme comprises several subthemes derived from consolidating 68 codes into 12 subthemes. The researchers translated the selected statements for each subtheme from vernacular Filipino to English, ensuring that the essence of the participants' original statements was retained throughout the paper, with only cultural nuances of the Filipino language being omitted. The succeeding tables show the source count, which indicates how frequently each theme appeared from the nine FGDs, while the reference count reveals how often these themes were mentioned throughout all the discussions.

**Table 3:** Major Themes

Themes	Source Count	Reference Count
Economic and Social Resources that Support or Hinder Participation	9	354
Self-Competency Affecting Transition	9	277
Flexible Service Delivery Mechanism	9	147
Safety and Security as a Threat to Participation	9	78

### Theme 1: Economic and Social Resources that Support or Hinder Participation

Theme 1 pertains to the resources that affect family members' participation in telerehabilitation sessions. It consists of two subthemes: engendered roles leading to parental burnout and resource accessibility unveiling the digital divide. (Table 4)

**Table 4:** Subthemes of Economic and Social Resources that Support or Hinder Participation

Subtheme	Meaning	Source Count	Reference Count	Selected Statement
Engendered Roles Leading to Parental Burnout	Other roles fulfilled by caregivers and parents compounded with online sessions lead to potential parental burnout.	9	354	“Mondays are tiring because there are modules and also online sessions. That's not the only thing I have to do, and I still have to do laundry, go to the market, cook rice, and cook food.” - *Participant 31*
Resource Accessibility Unveiling the Digital Divide	Economic and practical resources such as materials and internet connectivity affect participation in telerehabilitation sessions.	9	195	“When it's raining, we are affected because we just connect to the neighbor's internet connection, and when they remove it, we also get disconnected.” - *Participant 10*

The first subtheme, Engendered Roles Leading to Parental Burnout, discusses the roles and responsibilities of family members, including parents, grandparents, siblings of CWD, and therapists during telerehabilitation. Family members shared that with the shift to telerehabilitation, they became the primary providers of therapeutic intervention for their children. In addition to the sessions, they have various roles and responsibilities, such as teaching their children, doing household chores, and more. These responsibilities can hinder the success of telerehabilitation as they may be tiresome and overwhelming. The role of therapists facilitates success as they are skilled and flexible, especially when challenges are encountered during telerehabilitation. However, a parent stated that the sudden absence of therapists can be a barrier, making it more difficult for the parent to conduct the telerehabilitation sessions. Considering these factors, they could contribute to the eventual burnout of caregivers and parents, which could affect the roles being fulfilled and the child's telerehabilitation outcomes.

The second subtheme, Resource Accessibility, unveils the digital divide and includes internet connection, electrical power, financial constraints, and material and physical resources. They expressed how poor internet connectivity and electricity problems hinder their telerehabilitation sessions, as well as the lack of materials or the difficulty in gathering these when asked to prepare for activities given different obstacles. Some also expressed being unable to gather materials needed for the telerehabilitation session due to financial problems. Environmental factors, such as space availability and external noises or distractions, are considerations when conducting telerehabilitation.

### Theme 2: Self-Competency Affecting Transition

Theme 2 pertains to the preconceptions, experiences, and encounters the family members and the child experience while they adjust to telerehabilitation. It consists of four subthemes: Compliance and Performance of the Child under the Supervision of the Caregiver, Previous Online Experience, Educational Background affecting Learning Capacity, and Therapist guidance. (Table 5).

**Table 5:** Self-Competency Affecting Transition

Subtheme	Meaning	Source Count	Reference Count	Selected Statement
Compliance and Performance of the Child with the Parents or Caregiver.	Experiences of family members on children's behaviors as a factor to compliance in activities	9	256	“There was a time that my child did not want to follow. Maybe he's thinking, “She's not a teacher. She's not a therapist.” That's how 1 felt.” - *Participant 10*
Previous Online Experience	Perceptions of Telerehabilitation and previous experiences in the online setup as a factor in parent attitude and adjustment	9	202	“At first, he would start having tantrums, get unruly, and he does not want to attend therapy. However, when we started his online class in the afternoon, he got used to it. His siblings also attended online classes, so online activity seems normal for him.” - *Participant 5*
Educational Background Affecting Learning Capacity	The educational capacity of families is a limiting factor in implementing strategies at home	9	74	“As parents, we don't know much about doing therapy…but when we had the orientation, it seemed okay since there were instructions. What other techniques can make the child do the activities properly? Because it's difficult, especially for us, since we didn't study this.” - *Participant 27*
Therapist Guidance	Family members work together with therapists to implement telerehabilitation strategies effectively.	9	62	“With the guidance of the Kabahagi therapists, it becomes easier…if we are doing it correctly, we are guided by the therapists.” - *Participant 1*

The first subtheme, Compliance and Performance of the Child with the Parents or Caregiver, includes narratives from families expressing that their children are either compliant or hesitant in telerehabilitation sessions due to the unfamiliarity of the setup. Some parents reported that their children were less engaged and cooperative during therapy sessions due to the lack of in-person interaction with their therapists. Some families developed strategies to help their children ease in and comply during activities, such as routinely briefing them before the session to prepare them for what to expect and getting them accustomed to a routine schedule to anticipate the scheduled therapy session.

The second subtheme, Previous Online Experience, narrates the outlook of family members towards telerehabilitation and how their experiences with the online setup help with their telerehabilitation sessions. Initial skepticism was observed from parents' unfamiliarity with virtual therapy and concerns about its effectiveness. The caregivers questioned how online sessions could match the quality of in-person, hands-on therapy and worried about potential challenges they might face when implementing activities at home. Optimistic remarks were also expressed, especially gratitude for the opportunity for their child to receive services safely. These perceptions affect their participation during telerehabilitation sessions. Some expressed that their hesitation and adjustment were alleviated by their familiarity with the online setup, given that their children were already attending online classes.

The third subtheme, Educational Background Affecting Learning Capacity in telerehabilitation, includes sentiments from families about not being ready for remotely-implemented therapy sessions since this is a new setup that involves their full participation, unlike in-person sessions where the therapists directly cater to their children. Caregivers are also concerned about their lack of knowledge on how to perform therapeutic strategies that are virtually taught by the therapist, which leads them to question their capacity to execute the strategies properly. This affects family members' participation in continuing the strategies implemented during the sessions at home.

The fourth subtheme, Therapist Guidance, highlights the importance of family-therapist collaboration during telerehabilitation sessions to achieve therapeutic outcomes. Open communication between families and therapists emerged as crucial for successful telerehabilitation. Parents reached out to therapists for guidance on specific techniques and strategies, while therapists followed up with families to ensure they understood and could implement the activities discussed during sessions. Many families expressed gratitude for therapists who empathized with their challenges and provided ongoing support beyond the virtual sessions

### Theme 3: Flexible Service Delivery Mechanism

Theme 3 focuses on therapists' flexible approaches to delivering services during telerehabilitation. It includes flexibility in method, platform, and schedule. (Table 6).

**Table 6:** Subthemes of Flexible Service Delivery Mechanism

Subtheme	Meaning	Source Count	Reference Count	Selected Statement
Flexibility in Service Delivery Methods	Approaches like incorporating videos and images and implementing techniques to encourage adherence are utilized to complement synchronous sessions.	9	78	“They ask about my child's interests, which may be their way of focusing during our sessions. It is then incorporated into our therapy sessions, which my child looks forward to, along with the rewards in games. Her therapist also makes activities and presentations, which makes my child listen and comply with them.” - *Participant 12*
Flexibility in Platform	Use of various platforms such as Zoom, Facebook Messenger, Google Meet, and modes of communication such as phone calls, video calls, pictures, and videos	9	43	“I struggle with using my gadget since my phone is low-tech. Whenever I need to send files to the therapist, I cannot immediately send them since it requires a larger memory capacity. The therapist had to find another way for me to send her the files. She sent me her email. Also, there are times when I rented a computer to send the videos.” - *Participant 25*
Flexibility in Schedule	Strategies therapists use to compensate for missed synchronous sessions	8	40	“I am busy because of many things I do at home. I work from home and had doubts about whether we could do it. It's also my child's first time attending virtually, so we had to adjust a lot, and most activities became asynchronous. But the technique was that the teacher and I worked together. - *Participant 7*

The first subtheme, Flexibility in Service Delivery Methods, reveals that family members find that asynchronous sessions facilitate their learning during telerehabilitation by providing additional materials (videos and pictures) that can be quickly reviewed. They shared that the sessions were challenging to facilitate because their child was non-compliant. Hence, the therapists used and taught them using different methods to gain the interest and compliance of the child during telerehabilitation sessions, which included the use of positive reinforcements, like food, stars, stickers, rewards, and activities that the child enjoys.

The second subtheme, Flexibility in the Platform, indicates family members’ appreciation of using a variety of video conferencing platforms and gadgets that are convenient for them to facilitate telerehabilitation sessions. For some, this depends on the amount of available data allowance they have. Most family members use their phones to attend sessions, while others have a desktop or tablet available. Regarding platforms, most use Facebook Messenger as it is accessible, efficient, and easy to communicate with their therapist. Those with reliable internet connections used Zoom or Google Meet for synchronous sessions.

The third subtheme, Flexibility in Scheduling, highlighted how families occasionally missed sessions due to various challenges including internet connectivity issues, power outages, child non-compliance, errands, and work commitments. In response, therapists adapted by providing alternative solutions, such as sending instructional materials or demonstrations. Families would then record videos of their children performing the activities, allowing therapists to observe and provide feedback asynchronously.

### Theme 4: Safety and Security as a Threat to Participation

Theme 4 highlights critical concerns that compromise children's engagement and progress in telerehabilitation, particularly related to health vulnerabilities and concerning disciplinary practices. These threats not only jeopardize the child's immediate participation in therapy but also raise serious concerns about their physical and emotional well-being during virtual sessions. (Table 7).

**Table 7:** Safety and Security as a Threat to Participation

Subthemes	Meaning	Source Count	Reference Count	Selected Statement
Child's Poor Health	Children's illness could affect daily activities, the child's and family's well- being, and participation during telerehabilitation sessions.	8	37	“Lately, my child got sick. This is why we were not able to participate in online therapy because she had difficulty breathing and had coughs and colds.” - *Participant 37*
Maladaptive Discipline	Family members resort to punishment methods to facilitate the child's compliance, increasing abuse during telerehabilitation sessions.	4	6	“Sometimes 1 scare her. Sometimes, 1 have slippers beside me to scare her. ‘Finish your therapy session in 1 hour; it's just for an hour.” - Participant 28

The first subtheme, Child's Poor Health, reveals that when children become ill, with some being admitted to hospitals, they have to stop receiving regular therapy sessions, which leads to the eventual regression of the child as they are unable to continue the tasks and activities. Family members expressed that this caused them to go back to square one in terms of progress and made their current therapy sessions more difficult to facilitate as the child's skills or state may have been affected by the illness. One parent stated that due to the child being sick and unable to attend sessions, his body became stiffer, which increased the difficulty of the child's participation when they returned to attending their sessions.

The second subtheme, Maladaptive Discipline, pertains to the adverse methods caregivers use toward their children to bring about compliance during telerehabilitation sessions. Family members reported using methods such as frightening and threatening the child into obedience. One parent threatened a child with slippers if he deemed the child too unruly during the therapy session. In the Philippine culture, the use of corporal punishment is still widely practiced as a form of discipline. Some family members resorted to physical punishment as a means of enforcing discipline, unaware that such threats and abusive practices not only traumatize the child but also undermine their therapeutic progress. This forced compliance through fear is detrimental to both the child's well-being and their ability to engage meaningfully in therapy.

## Discussion

The shift in the delivery of healthcare services has positioned telerehabilitation as an alternative to in-person therapy sessions. In the Philippines, the increased access to information and communication technologies (ICTs) has facilitated the rise of telerehabilitation, offering advantages not only for patients and families but also for healthcare workers (Macariola et al., 2021). As the demand for healthcare services is expected to grow due to population growth (Raghavan et al., 2021), telerehabilitation is seen as a potential solution to future challenges brought about by factors that hinder access to in-person health services. However, healthcare professionals should consider the context of families participating in telerehabilitation (Boelsma et al., 2021). It is crucial to consider the inclusion and rights of children with disabilities and the role of family members in the implementation, especially among the urban poor communities.

### Challenges and Opportunities in Telerehabilitation

The transition to telerehabilitation has presented significant challenges for families of children receiving therapy services. Research indicates that parents often express skepticism about telerehabilitation's effectiveness, particularly regarding treatment outcomes, home implementation of therapeutic strategies, and communication with healthcare providers (Lam et al., 2021). A notable concern is the observed decrease in children's compliance during initial virtual sessions compared to traditional in-person therapy, where therapists can directly assist parents in behavior management.

To address these challenges, literature suggests several evidence-based strategies. Cohen et al. (2018) recommend incorporating visual aids such as pictures, puppets, and toys while emphasizing the importance of simplified communication and measured pacing during sessions. Additionally, parents must invest considerable time and effort in managing both technological aspects and their child's behavior throughout virtual sessions (Grillo, 2017; Yoo et al., 2020). Although this adjustment period may initially seem daunting, research demonstrates that parent-implemented interventions can effectively enhance children's language and communication outcomes (Akamoglu & Meadan, 2018; Camden et al., 2019).

This transition to telerehabilitation presents an opportunity for clinicians to empower families with essential skills and knowledge for home-based therapy implementation. Parenting quality is one of the most important factors on the child's development (Shapiro, 2013). By providing targeted parent education and support programs, therapists can equip families with specific behavior management strategies, reward systems, and compliance techniques tailored to their child's context and interests. Nevill et al. (2016) affirm that individualized parent training can facilitate children's skill development. Through this collaborative approach, therapists can enhance both child participation and therapeutic outcomes while supporting parents in achieving their intervention goals.

### Family-Centered Approach and Collaboration

The implementation of telerehabilitation has highlighted the significance of family-centered practice, where families demonstrate increased engagement and motivation through shared therapeutic activities (Lambert et al., 2021). While this collaborative approach enhances therapeutic fellowship, it's important to acknowledge that families often experience pressure, fatigue, and overwhelming feelings due to multiple roles and responsibilities that may impact service delivery until alternative solutions are developed (Hao et al., 2021). To support sustainable family engagement and prevent parental burnout, practitioners must be prepared with appropriate strategies (Griffith, 2020) and focus on educating families about adapting to virtual or hybrid setups while creating optimal therapeutic environments.

The family-centered nature of Community-Based Rehabilitation emphasizes the critical importance of family-therapist collaboration in promoting child advocacy and maintaining commitment to children's needs while fostering partnerships that honor the unique strengths, cultural perspectives, and expertise each member brings to treatment planning (Kokorelias et al., 2019). This collaborative approach has shown positive outcomes, with telerehabilitation facilitating increased family member participation (Bate & Malberg, 2020) and parents reporting enhanced communication support skills (Lincoln et al., 2015). To strengthen these partnerships, policymakers and healthcare professionals should prioritize consistent family-therapist communication from initial sessions while developing joint strategies that consider family circumstances and providing educational resources to empower families as essential partners in the therapeutic process (Banks et al., 2017, cited in Longo et al., 2020).

### Addressing Economic and Practical Barriers

The successful implementation of telerehabilitation faces significant economic and technological barriers, particularly in developing countries like the Philippines, where families experience increased pressure to acquire information and communication technology (ICT) resources for virtual therapy sessions (Macariola et al., 2021). Critical challenges in Community-Based Rehabilitation include poor internet connectivity, inadequate cellular reception, technological literacy barriers, and limited access to appropriate devices (Chivate et al., 2022).

While video conferencing platforms have become more accessible to practitioners and clients (Weerathunge et al., 2021), the quality and consistency of therapy sessions remain heavily dependent on families' access to reliable internet connections, sufficient data allowance, appropriate devices, and technological proficiency. Facebook Messenger emerged as the preferred platform due to its accessibility and user-friendly interface, though resource limitations in urban poor communities continue to impact service delivery. To address these challenges, service providers have implemented adaptive strategies such as asynchronous sessions and rescheduling options. Additionally, the provision of printed booklets has proven essential, particularly for families with lower educational levels in low- and middle-income countries (Banks et al., 2017 cited in Longo et al., 2020).

The findings of this study are aligned with research that families value the adaptability of telerehabilitation systems that accommodate their available technologies (Lambert et al., 2021). To enhance the effectiveness and acceptability of telerehabilitation programs, particularly in resource-constrained environments, service providers must carefully consider planning processes and establish clear lines of communication (Lincoln et al., 2015). This systematic approach to addressing economic and practical barriers is crucial for ensuring equitable access to telerehabilitation services.

### Addressing Safety and Security Concerns

The emergence of telerehabilitation has heightened the need to address safety and security concerns, particularly regarding disciplinary practices and child protection. The global implementation of stay-at-home orders during the pandemic has been associated with increased incidences of domestic violence (Kourti et al., 2021), raising significant concerns about the safety of children with disabilities. As telehealth transitions from an alternative to a standard form of service delivery, outlined in Advisory No. 2021-001, released by the Philippine Academy of Occupational Therapists (PAOT, 2020), healthcare professionals and policymakers must establish robust protocols to identify and address potentially harmful practices. This includes developing comprehensive family education programs about positive disciplinary approaches and their impact on child well-being while implementing systematic monitoring procedures for children deemed at risk (Ertan et al., 2020).

### Policy Implications and Recommendations

The widespread adoption of telerehabilitation necessitates comprehensive policy reforms and funding mechanisms to address systemic barriers in service delivery, particularly for children with disabilities from low-income families. Research indicates that technological challenges and connectivity issues remain primary obstacles, suggesting the need for public programs and financial assistance schemes to support families facing these difficulties (Kruse & Heinemann, 2021). Effective policy development requires a multi-level approach encompassing personal, organizational, and governmental spheres, emphasizing cooperation between government and community stakeholders to ensure successful implementation (Fisher & Purcal, 2016).

International frameworks, such as Article 11 of the Convention on the Rights of Persons with Disabilities, provide valuable guidance for local legislators in developing inclusive policies that recognize persons with disabilities as essential partners in humanitarian response and emergency situations (Motz, 2018). This is particularly crucial given that children with disabilities remain among society's most vulnerable populations, often experiencing inequality, dignity violations, and autonomy deprivation. While initiatives like the Philippine Commission on Human Rights advisory series address disability rights (Commission on Human Rights, 2019), current policies tend to overgeneralize the needs of persons with disabilities, and their practical impact remains limited (Velasco et al., 2021). These challenges underscore the need for more nuanced, targeted policy approaches that prioritize expanded insurance coverage for telerehabilitation services, establish sustainable funding mechanisms for assistive technology acquisition, and develop comprehensive financial assistance programs for low-income families accessing virtual therapy services.

## Conclusion

In conclusion, this study has highlighted the pressing need to address the challenges faced by CWD from low-income families in accessing community-based telerehabilitation. To ensure equitable access to quality care, it is crucial to implement targeted interventions and support systems that overcome the unique barriers encountered by this vulnerable population. Actionable strategies should focus on alleviating economic and practical barriers by providing affordable or subsidized technology and internet access and offering training and support to families to enhance their digital literacy and ability to assist their children during therapy sessions. Developing culturally sensitive and linguistically appropriate resources and materials is essential to support family-centered collaboration. Furthermore, establishing clear protocols and guidelines for ensuring the safety and well-being of CWD during telerehabilitation sessions, including measures to prevent and address instances of abuse or neglect, is of utmost importance. Advocating for policies and funding mechanisms prioritizing CWD needs from low-income families, such as expanded insurance coverage for telerehabilitation services and financial assistance programs, is critical to building a more inclusive and resilient healthcare system. By implementing these actionable points, community-based telerehabilitation can work towards empowering CWD from low-income families and their caregivers to access the care and support they need to thrive, ensuring that no child is left behind.

## Limitations and Recommendations

This study did not explore the effectiveness of the methods of service delivery nor perspectives of the service providers and is limited to the experiences of families who received ten sessions of free telerehabilitation services. Given the heterogeneous nature of device availability, future researchers are encouraged to investigate the experiences of family members and how the accessible technology influences the quality of services received. This study did not examine the impact of varying device specifications (e.g., screen dimensions, type) on the effectiveness of interventions administered to participants. While most participants demonstrated proficiency with Facebook's messaging application, potential data privacy concerns may arise due to its connection to personal accounts containing diverse personal information.

Additionally, comparative studies of online session outcomes across various rehabilitation science disciplines and assessing their effectiveness is warranted. Further research is recommended to evaluate the economic feasibility and cost implications of this service delivery modality, as these factors significantly influence sustainability and long-term implementation.
